# Nucleo–cytoplasmic transport defects and protein aggregates in neurodegeneration

**DOI:** 10.1186/s40035-020-00205-2

**Published:** 2020-07-03

**Authors:** Giacomo Bitetto, Alessio Di Fonzo

**Affiliations:** IRCCS Foundation Ca’ Granda Ospedale Maggiore Policlinico, Dino Ferrari Center, Neuroscience Section, Department of Pathophysiology and Transplantation, University of Milan, Via Francesco Sforza 35, 20122 Milan, Italy

**Keywords:** Aging, Neurodegeneration, Neurodegenerative disease, Nucleo–cytoplasmic transport, Nuclear pore complex, Protein aggregate

## Abstract

In the ongoing process of uncovering molecular abnormalities in neurodegenerative diseases characterized by toxic protein aggregates, nucleo-cytoplasmic transport defects have an emerging role. Several pieces of evidence suggest a link between neuronal protein inclusions and nuclear pore complex (NPC) damage. These processes lead to oxidative stress, inefficient transcription, and aberrant DNA/RNA maintenance. The clinical and neuropathological spectrum of NPC defects is broad, ranging from physiological aging to a suite of neurodegenerative diseases. A better understanding of the shared pathways among these conditions may represent a significant step toward dissecting their underlying molecular mechanisms, opening the way to a real possibility of identifying common therapeutic targets.

## Background

Neuronal protein aggregates are a characteristic neuropathological hallmark of neurodegenerative disorders such as amyotrophic lateral sclerosis (ALS), frontotemporal dementia (FTD), Alzheimer’s disease (AD), Huntington’s disease (HD), polyglutamine expansion related ataxias, and Parkinson’s disease (PD) [1]. One possible consequence of these aggregates is interference with the proper functioning of the highly conserved nucleo–cytoplasmic transport (NCT) mechanism in the cell, which ensures the transport of nucleic acids and proteins across the nuclear membrane. This pathway is fundamental for long-lived cells such as neurons [2, 3], and emerging evidence links damage to the nuclear membrane and nuclear pores to neurodegenerative diseases and physiological neuronal aging [4].

A properly functional NCT is fundamental for neurons to allow transcription factors to enter into the nucleus [5]. One essential NCT role is to activate regulatory elements necessary for adaptation and response to external stimuli, especially those implicated in neuronal plasticity [6]. Moreover, in non-dividing post-mitotic cells such as neurons, proteins involved in DNA maintenance and repair reach the nucleus through this mechanism [7, 8]. In addition, all ribonucleic proteins involved in processes of RNA maturation, stability, splicing, and export require this transport for localization to the nucleus [9].

The cell relies on an essential, conserved, and dynamic structure called Nuclear Pore Complex (NPC) to accomplish a correct NCT [5]. Small molecules (molecular weight < 40 kDa, diameter < 5 nm) can relatively freely diffuse through the NPC [5]. In contrast, higher molecular-weight proteins depend on the highly regulated and very specific mechanisms of the NCT machinery [5].

NPCs are large cellular structures that span the nuclear envelope [10] and control exchanges between the cytoplasm and nucleus. The NPC is constituted by proteins collectively referred to as nucleoporins (Nups) [11] that have different roles in regulating NPC functions and transport of matter, energy, and information between the nucleus and cytoplasm [12]. In addition, the NPC is involved in cell cycle regulation, chromatin organization, and gene activation [6, 13].

Despite the relatively large size of NPCs, most proteins and RNAs cannot freely diffuse through them. Indeed, the passage through NPC is a highly coordinated and selective process, mediated by a family of soluble receptors called karyopherins (Kaps), also known as importins/exportins [14]. The small Ras-related nuclear protein (Ran) plays a central role in this transport and regulates interactions between Kaps and their cargos on either side of the NPC [15]. Ran is the only known member of the Ras superfamily of small GTP-binding proteins that is localized principally inside the nucleus [16, 17]. A gradient of RanGDP/GTP across the nuclear membrane is essential in establishing the directionality of the transport. To maintain the gradient, RanGTP is hydrolyzed to RanGDP on the cytoplasmic side and RanGDP is converted to RanGTP in the nucleus [17].

The nuclei of human cells have several thousand NPCs, with a structure that seems to have been highly conserved, underlining their importance for cells [18]. Nevertheless, despite this high degree of structural conservation of the NPC complex, two crucial time points in evolution from prokaryotes to humans have been suggested in NPC development, implying that the nuclear pore is still highly adaptive and flexible at the sequence level [19].

Neurons seem particularly sensitive to the damage of these structures, as demonstrated by the exclusively neurodegenerative consequence of many diseases due to damage of the NPC function and the NCT. So far, several reasons for this vulnerability have been studied. One clue is the inability of neurons to undergo mitosis, the usual process of cellular renewal [20]. Through DNA replication, cells maintain physiological protection of the genome from exogenous damages. To preserve genomic integrity, there are at least four active DNA repair pathways in nervous system each corresponding to a particular type of DNA lesions [21]. Genome instability can appear when the accumulation of DNA damage exceeds a neuron’s repair capacity or the DNA repair machinery is defective [20]. Thus, transport across the nuclear membrane represents a unique possibility for neurons to guarantee DNA integrity, and genome instability is a major factor in neuronal aging [22, 23]. For example, somatic single nucleotide variants have been reported to be in excess in neurons from people affected by early-onset degeneration with DNA repair gene mutations [24].

Cellular oxidative stress is one of the primary sources of DNA damage, mostly in the brain due to its high demand for energy and increased radical oxygen species (ROS) formation [25]. This process, in addition to direct effects on DNA stability, is increasingly reported to affect NCT [26, 27]. At least four mechanisms have been associated with oxidative stress: reduced Ran GDP/GTP ratio, mislocalization of Nups, altered functions (binding, docking) of importins/exportins, and decreased integrity of the nuclear membrane [28].

RNA transport defects are an additional sign of neuronal sensitivity to NCT impairment, and of course, the export of RNAs from the nucleus to the cytoplasm is key to gene expression [29]. Moreover, a tRNA retrograde transport between cytoplasm and nucleus has recently been proposed as part of the cellular response to oxidative stress [30]. Among neurons, proper RNA shuttling function appears even more relevant for cells with a high transcriptional activity such as Purkinje cells and motor neurons [31–33]. All of these NCT defects that significantly affect DNA and RNA are related to aging and are even more relevant in neurodegeneration [26]. RNA-binding proteins mutated in specific neurodegenerative disorders (TDP-43, FUS, and hnRNPA1) can alter the dynamics of membrane-less organelles such as stress granules, or the more recently identified liquid droplets, and accelerate fibrillization in neurons, resulting in the formation of pathological amyloid-like fibrils that deposit in the cell bodies and neuropil [34–36].

**Fig. 1:**
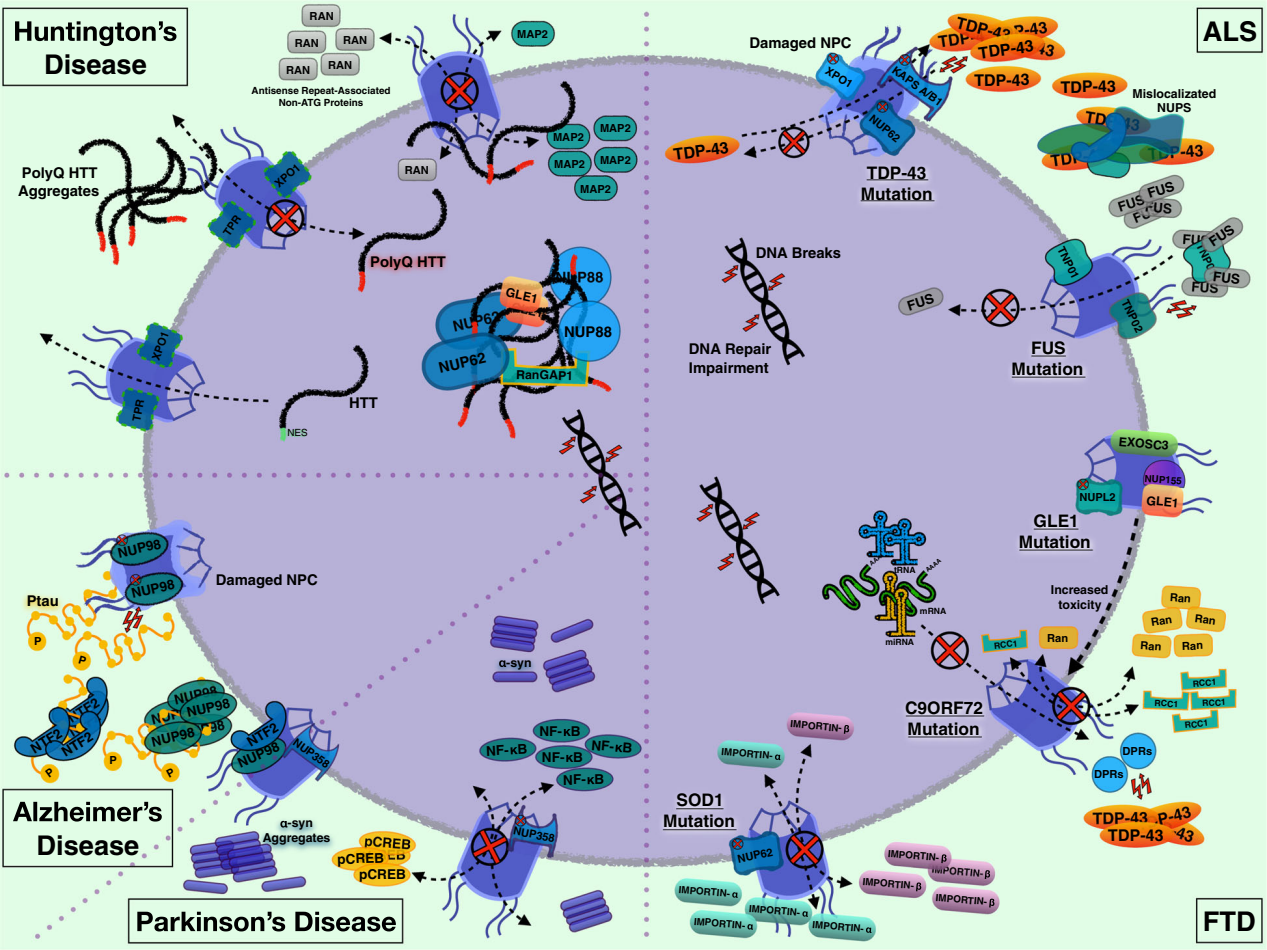
Representation of the nucleus and NPCs with Nups, protein/RNA aggregates in several neurodegenerative diseases. ALS/FTD: - Damaged NPC and specific Nups involved in the presence of TDP-43 cytoplasmic aggregates and impaired TDP-43 nuclear import. - Impaired FUS import in the presence of aggregates containing FUS with the importin TNPO1 or alone. - Impaired RNA export in *C9ORF72* mutations with DPRs and TDP-43 protein aggregates formation. - Altered Ran and RCC1 nucleo-cytoplasmic distribution; C9ORF72 toxicity is increased by GLE1. - EXOSC3 dysfunction. - Altered distribution of importin-α and -β in *SOD1* mutation with Nup62 impairment. HD: HTT physiological transport across the NPC through the interaction of NES with TPR and XPO1. Aberrant shuttling of RAN proteins and MAP2 due to PolyQ HTT affecting the NPC. Intranuclear aggregates of PolyQ HTT sequestering Nup62, Nup88, GLE1, and RanGAP1. AD: Phospho-tau aggregates induce NPC damage and accumulation of NTF2 and Nup98 in the cytoplasm; Nup98 loss, in turn, may facilitate tau aggregation. PD: Cytoplasmic aggregates and intranuclear alpha-synuclein in Parkinson’s disease; pCREB aggregates and nuclear accumulation of NFkB are associated with NPC and Nup358 defects in PD

Neurodegenerative disorders display a unique pathological hallmark arising from specific gene mutations. This hallmark is protein aggregates, which are known to damage NPCs [34, 37, 38], and evidence indicates that protein and RNA aggregates may interfere directly with specific Nups [34, 37]. In some neurons, Nups mislocalization and altered NPC function could lead to an even greater increase in aggregate accumulation and genome stress due to chromatin and DNA/RNA impairment [39, 40]. Further complicating this picture, the pathogenic process includes aberrant transcript maturation, transport, and translation [41] (Fig. 1).

The neuronal inclusions detected in neurodegenerative forms have been linked to deficient mechanisms of degradation, especially proteasomal and autophagy processes [42].

Whether the pathological cascade, starting from the formation of the aggregates and the damage of the NPC, to the enhanced fibrillation, may represent a common paradigm underlying different neurodegenerative diseases is still unknown.

In summary, several lines of evidences are emerging to suggest a link between the formation of neuronal aggregates and the structural and functional damages of the NPC, as well as the NCT pathways. However, it is not clear yet whether NCT dysfunction act as an upstream common pathogenetic mechanism in the neurodegenerative process or a downstream event triggered by specific pathological aggregates of different neurological disorders. To give an overview that may facilitate the design of future studies, we review here the most relevant pieces of evidence of NCT impairment in neurodegeneration. We explore several neurodegenerative diseases, their pathogenic mechanisms, and genetic causes, highlighting the role of NPC and NCT as key factors in modulating the neurodegenerative process and also physiological aging.

## Main text

### NPC structure and function

The nucleus is the central and distinguishing organelle of eukaryotic cells, encompassed by a double membrane dynamic structure called the nuclear envelope [43]. This envelope consists of an outer membrane that is directly continuous with the rough endoplasmic reticulum and an inner membrane that contains a specific set of nuclear envelope transmembrane proteins [44]. Internal to the nuclear envelope is the nuclear lamina, a dense fibrillar network of intermediate filaments that surrounds the cellular genome [45]. These structures guarantee the specific eukaryotic compartmentalization that segregates the DNA from the cytoplasm. Accomplishing this function requires an accurate system providing proper communication and molecular transport among the different cellular compartments [46, 47], which is the role of NPCs.

Vertebrate NPCs are ∼70 nm protein channels spanning the nuclear envelope, with a cylindrical scaffold of ∼125 nm and internal diameter of ∼40 nm [48]. The channel connects the nucleus and the cytoplasm. NPC is the largest cellular protein structure at ∼125 MDa [12], consisting of more than 30 different proteins called Nups [49, 50]. When assembled, they form a cytoplasmic ring, spoke ring, and nuclear ring [12]. Eight filaments are attached to the rings at the nuclear and cytoplasmic sides [51–53]. On the nuclear side, ∼50 nm filaments are connected in a basket-like structure, while on the cytoplasmic side, the filaments are linked to the Nup214 complex [9, 48, 54, 55].

The NPC has a complex and highly regulated function, and most of the specific roles of single Nups are not well known. What is known is that a particular group of Nups in the central channel is fundamental to the selective barrier and substrate-specific transport role of NPCs [56, 57]. These Nups are characterized by phenylalanine-glycine (FG) domains and are anchored to the core scaffold through linker Nups [58]. Moreover, several Nups seem to exhibit a certain level of redundancy and functional overlap, forming an extremely dynamic barrier [56, 57].

With their intrinsic disordered FG domains, FG Nups form a dynamic filter that prevents passive diffusion of molecules through the NPC and allows for regulated transport of larger protein complexes of up to 40 nm [59, 60]. A single pore can contain 6 MDa of FG repeats, providing docking sites for import and export nuclear transport receptors (NTRs) that are crucial for selective passage [54, 61]. Molecules passing from the cytoplasm to the nucleus and vice versa must bear specific signaling sequences to interact with the NPC [54]. NPCs do not change from a defined “closed” to an “open” state during this passage, and the bond with the substrates consists of multiple weak contacts between the NTRs and the FG Nups assembly [58, 62, 63].

Among the many different NTRs, the largest group is the highly conserved family of proteins known as Kaps, consisting of more than 20 members (importins, exportins, and transportins) in humans [64]. Kaps can transiently interact with Nups and their FG domains to facilitate the shuttling of specific macromolecules through the NPC [64]. Motifs adjacent to FG repeats coordinate termination of transport and release of transporting complexes from the NPC [65].

Only a few other Kaps, such as importin-α, importin-β1, importin-β2, and chromosome region maintenance 1 (CRM1), have been well characterized [66, 67]. The same is true for the interactions among these proteins, with only a few well elucidated, such as the link between importin β1 and Nup153 at the nuclear face of NPCs [44, 68]. Many transport receptors, e.g., importin-β and NTF2, have hydrophobic binding sites on their surface for these FG-Nups [69, 70].

The NTRs bind short signaling peptides presented on their substrates for nuclear import and export. These signaling regions have been generally referred to as nuclear localization signals (NLSs) and nuclear export signals (NESs). The link established between the receptor and the shuttled substrate can be direct or mediated by additional adaptors, such as importin-α itself between importin-β and cargoes [71].

The processes of shuttling cargo–receptor complexes into the nucleus or into the cytoplasm do not directly use energy from ATP/GTP hydrolysis but depend strictly on the small GTPase Ran [72–74]. The nucleoporin Nup358 (RanBP2) has four domains that can bind Ran [72]. This member of the protein Ras superfamily exists in two nucleotide states, bound to nucleotide guanine triphosphate (RanGTP) or to guanine diphosphate (RanGDP) [73, 74]. The intrinsic GTPase activity of Ran is low, but the GTPase-activating protein RanGAP1 [75] catalyzes the shift between the two different forms, from cytosolic RanGTP to RanGDP [76, 77].

In contrast, the Ran guanidine exchange factor RCC1 (Regulator of chromosome condensation 1) in vertebrates can promote the exchange of guanine nucleotides by RanGDP to RanGTP in the presence of GTP [75]. This process leads to a higher nuclear concentration of RanGTP in the nucleus. A continuous supply RanGDP from the cytoplasm to the nucleus is accomplished by nuclear transport factor 2 (NTF2) [78]. RCC1 only can be found attached to nuclear chromatin to guarantee unequal distribution of GTPase Ran states, as the presence of a RanGDP/GTP gradient across the nuclear envelope gives directionality to the transport [79].

**Fig. 2:**
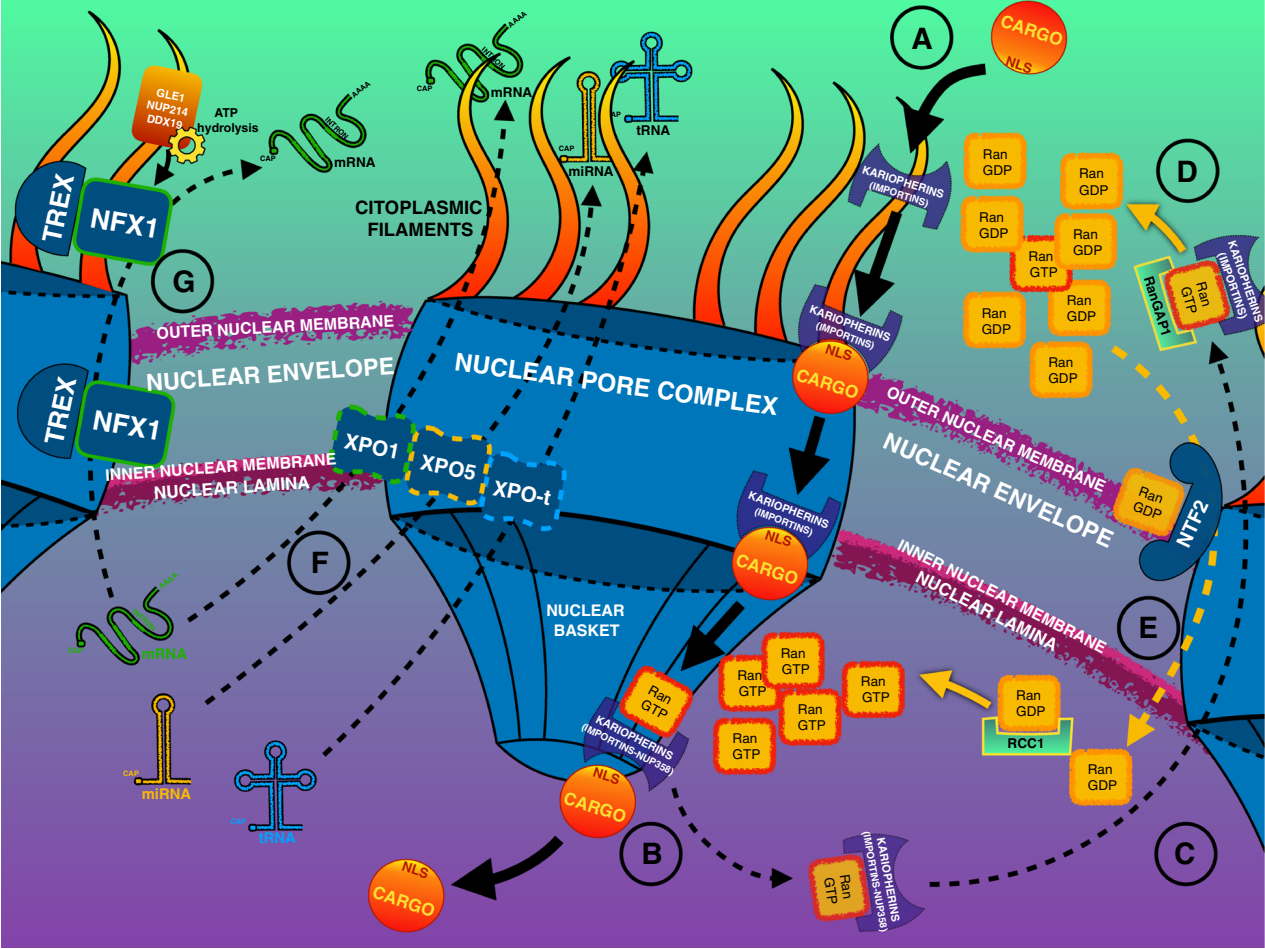
Representation of the NPC structure highlighting the import–export function of proteins and transcripts. **a** Cargo binds importins to be shuttled to the nucleus. **b** Nuclear RanGTP induces dissociation of the importin–cargo complex. **c** The importins bound to RanGTP are recycled to the cytoplasm. **d** RanGAP1 hydrolyzes RanGTP, maintaining the RanGDP/GTP gradient across the nuclear membrane. **e** RanGDP is imported into the nucleus through NTF2 and converted to RanGTP by RCC1. **f** RNAs are shuttled to the cytoplasm, binding their specific exportins. **g** mRNAs only may undergo an alternative NPC transport interacting with the NFX1–TREX complex. GLE1, Nup214, and DDX19 act as NFX1 modifiers to release mRNAs into the cytoplasm

In summary, importins can recognize their cargo in the cytoplasm, where the RanGTP concentration is low, and then bind to the Nups to go through the NPC. In the nucleus, RanGTP stimulates the separation of the complex and cargo release. The importin–RanGTP complex is recycled, and the RanGTP becomes RanGDP in the cytoplasm through the activity of RanGAP. In contrast, exportins act in the nucleus only in the presence of RanGTP, forming a trimeric cargo–receptor–RanGTP complex that, once transported in the cytoplasm, undergoes dissociation (Fig. 2a–e).

NPCs are essential not only for protein shuttling across the nuclear envelope but also for RNAs that need to be exported into the cytoplasm. However, the detailed mechanism of RNA transport is not yet fully solved [80]. Several NTRs are implicated, and the directionality is not often accomplished through the RanGDP/RanGTP gradient but by specific RNA helicases [81].

For example, exportin-t (XPO-t) and exportin 5 (XPO5) have key roles in shuttling specific RNAs. The former is implicated in the export of tRNAs, and the latter in the shuttling of double-stranded RNAs and pre-miRNAs [82, 83]. They use an RNA hairpin structure as an NES in a Ran-dependent process [84, 85]. A small subset of mRNAs use CRM1 (XPO1) for their export through the NPC [86]. The principal mRNA export receptor is NXF1 (Nuclear export factor 1) [87], which binds the mRNA in combination with several adaptors (THO complex, UAP56, and AlyRef), collectively forming the TRanscription and EXport complex (TREX) [88]. NTF2 mediates the interaction between NXF1 and the FG-Nups [89]. Once on the cytoplasmic side, Nups such as Gle1, Nup214, and the DEAD-box protein RNA helicase DDX19, that use ATP hydrolysis, revert NXF1 to a lower affinity RNA-binding state to release mRNAs [90]. The proteins involved in mRNAs shuttling are intricately linked with the mRNA modifications at several stages, including transcription, processing, splicing, and poly-adenylation (Fig. 2f-g). All these mRNA maturation processes and NCT components require a tight orchestration to ensure a correct cellular functioning.

### Neurodegenerative diseases

#### Amyotrophic lateral sclerosis and frontotemporal dementia

ALS, also known as Lou Gehrig’s disease, is a fatal neurodegenerative disease characterized by progressive loss of motor neurons in the brain and spinal cord [35]. ALS patients frequently present with behavioral and cognitive deficits, which are symptoms associated with atrophy in the frontotemporal cortex [91]. This particular degeneration had traditionally been linked to another disease, FTD, the most common cause of dementia after AD. Similarly, FTD patients can show motor neuron disease symptoms commonly associated with ALS [92, 93]. This duality prompted consideration of ALS and FTD as two ends of a spectrum disorder termed ALS/FTD.

Although only a small proportion of cases (10%) with ALS/FTD are familial, genetic factors have been firmly established as causative in both familial and sporadic cases, as several mutations display a low penetrance [35]. Mutations in several genes are widely accepted as relevant in ALS, FTD, or ALS/FTD, including *SOD1*, *TARDBP*, *FUS*, *C9ORF72*, *MAPT*, *TBK1*, *BCP*, *GLE1*, *OPTN*, *UBQLN2*, *SQSTM1*, *ANG*, *TUBA4A*, *MATR3*, *VCP*, and *CHCHD10* [35].

ALS and FTD cases with different monogenic forms present distinctive molecular neuropathologic signatures. The findings range from inclusions of TDP-43 to FUS and TAU to no inclusions of these proteins, as observed in patients with *SOD1* mutations [94]. In post-mortem brains of most patients with sporadic ALS/FTD (97% of ALS and 50% of FTD) [95], TDP-43 and FUS show an altered subcellular localization and appear to be at least partially lost from the nucleus in neuronal and glial cells [94].

Moreover, substantial evidence implicates oxidative stress as a central mechanism of motor neuron death in ALS [96].

##### TDP-43–related pathology

*TARDBP* encodes the predominantly nuclear RNA-binding TDP-43, a protein with a NLS and a “putative” NES that can shuttle between the nucleus and cytosol [97]. In addition to nuclear functions, TDP-43 is involved in multiple processes in the cytoplasm, including mRNA stability, transport, and translation [98]. TDP-43 is essential for neuronal survival and selective neuronal degeneration appears in the presence of pathological TDP-43 [99, 100]. The precise mechanism by which TDP-43 passes through the nuclear membrane is unclear; however, recent evidence suggests that despite the predicted NES, the protein is exported independently of the export receptor CRM1/XPO1 [101–103]. Thus, no single exporter is necessary for TDP-43 export and TDP-43 nuclear egress seems to follow a model of passive diffusion; these findings, provided by silencing specific Nups in Hela cells, suggest that redundant pathways regulate its transport [101, 102]. Other factors may influence TDP-43 cytoplasmic translocation, as recently demonstrated for the tumor-suppressor folliculin protein, which directly interacts with the RNA-recognition motif domain [104]. These experiments, performed in non-neuronal cell lines and in rat primary cortical neurons, leave unexplored the possibility of other mechanisms specific of human neurons, this should be investigated in future experiments [102].

Whether the TDP43 pathology causes import–export defects or whether defects in NPC components precede the TDP43 pathology is still unclear. TDP-43 is imported into the nucleus by the import pathway Kaps α/β1, using the Ran GDP/GTP gradient; “Kap α” is recycled to the cytoplasm via cellular apoptosis susceptibility protein (CAS) [105]. Disruption of this pathway in human and mouse neuroblastoma cell lines and primary murine cortical neurons, by downregulation of Kap β1 or CAS, leads to TDP-43 cytoplasmic accumulation [105]. Of note, some studies have shown decreased levels of CAS, Kap α2, or α6 in motor neurons of FTD patients with TDP-43 mutations and in ALS patients [105]. Direct evidence of TDP-43 translocation to the cytoplasm was nicely demonstrated in vivo in zebrafish, with an innovative fluorescent tool that visualizes the dynamic of protein transport in neurons [106]. In particular, TDP-43 reaches the cytoplasm, dendrites, and extracellular matrix, inducing neuronal cell death, and can form cytoplasmic aggregates in the absence of microglia [106].

However, if TDP-43 translocation after UV-mediated injury affects also other neuronal subtypes is still unexplored. Future studies aimed at filling this gap might help to clarify the mechanisms of motor neuron pathology selectivity in ALS/FTD.

TDP-43 pathology causes the mislocalization and aggregation of Nups and transport factors, as well as disruption of nuclear membrane and NPCs, leading to impaired nuclear protein import and mRNA export [34]. Differently, nuclear alterations of Nup62 or Kap β1 have been detected by nuclear staining of spinal motor neurons from ALS patients [107]. Recently, new emerging observations show that ribonucleoprotein complexes, such as those containing TDP-43, form liquid droplets with different biophysical characteristics at different neuronal sub-cellular locations [36, 108]. Interestingly, mutations of TDP-34 may confer toxic properties to these droplets [108]. Furthermore, TDP-43 was shown to undergo phase-separation into liquid droplets in the cytosol in vivo after TDP-43 overexpression or exposure to amyloid-like fibrillar TDP-43 or FUS [36]. Droplets of cytosolic TDP-43 were shown to recruit importin-α and Nup62, and to induce mislocalization of RanGap1, Ran, and Nup107, with the consequent inhibition of NCT [36]. Further experiments in this promising direction will define the intricate interplay between the role of pathological aggregates and the alteration of Nups and consequently of the NCT, perhaps also taking into consideration other neurodegenerative pathologies characterized by alterations of different ribonucleoproteins.

Experimental models using Neuroblastoma cell lines overexpressing several Nups and wild-type or mutated TDP-43 have shown that FG repeat-containing Nups, scaffold Nups, and nuclear export factors co-aggregate with mutated TDP-43. Nup205 has been found to mislocalize in patients’ fibroblasts, induced pluripotent stem cell (iPSC)-derived motor neurons, and showed co-aggregation with TDP-43 positive cytoplasmic inclusions in brain tissue of ALS patient [34]. Starting from these observations, future studies evaluating additional Nups mislocalization and co-aggregation with TDP-43 in ALS and FTD brain samples and iPSCs-derived motor neurons will define a clearer picture of the neurodegenerative process involving NPC and protein aggregates. In the same study, NPC defects in the *Drosophila* model expressing human mutant TDP-43 could be rescued by reducing TDP-43 aggregates and vice versa, suggesting a complex interplay of both components of the degenerative process [34]. In ALS/FTD fly models with the hexanucleotide *C9ORF72* repeat expansion, accumulated cytosolic TDP-43 is found to cause Kap α2/ α4 pathology, to increase levels of dipeptide repeat proteins (DPRs), and to enhance expansion-related toxicity [109].

Finally, actin polymerization was recently linked to NPC integrity and function. Modulation of actin homeostasis in primary motor neurons seems to rescue NPC instability arising from mutant *Profilin1* and *C9ORF72* repeat expansion [110].

##### FUS-related pathology

The term “FUSopathies” designates neurodegenerative diseases characterized by neuronal and glial cytoplasmic fused in sarcoma (FUS) inclusions [111]. Cytoplasmic aggregates of FUS can be found in 5% of familial ALS and up to 10% of ALS/FTD brains. Mutations in the FUS gene occur in 5% of familial ALS and < 1% of sporadic ALS cases, but are rare in FTD [112–114].

FUS is a predominantly nuclear protein with DNA and RNA binding properties involved in regulating transcription, splicing, miRNA biogenesis, and RNA transport [115, 116]. Among its functions, FUS is also essential for DNA repair, being recruited by PARP1 at DNA damage sites [117, 118]. It belongs to the family of RNA-binding proteins known as FET (FUS/TLS, EWS, and TAF15). At their C-terminus, FET proteins have a homologous PY-NLS motif interacting with β-Kaps TNPO1 or TNPO2, which are involved in nuclear import [119, 120]. FUS was seen to translocate through a calcium-dependent mechanism, and NCT alterations result from its cytosolic translocation [121]. The majority of ALS-associated FUS mutations affect the C-terminal NLS, interfering with TNPO1 binding and consequently leading to FUS cytoplasmic accumulation [122]. Post-translational modifications of FET proteins can trigger the same outcome, affecting their interaction with TNPO1 [123].

FUS inclusions in ALS and FTD differ in that FUS is methylated in ALS and not methylated in FTD. In the first case, TNPO1 does not co-localize with the inclusions, and FUS is not properly imported into the nucleus. In the second, FUS inclusions are not methylated, and TNPO1 co-localizes with FUS inclusions, implicating other factors in cytoplasmic accumulation of the protein [123, 124].

Several lines of evidence suggest a major role of DNA repair impairment resulting from FUS mutations [125, 126]. Loss of nuclear FUS causes DNA nick ligation defects in motor neurons because of reduced recruitment of XRCC1/LigIII to DNA strand breaks, a mechanism that also has recently been associated with AOA4 [125, 126].

In iPSC-derived motor neurons, the induced DNA damage leads to FUS mislocalization in the cytosol, enhancing a vicious cycle by increasing cytoplasmic FUS shuttling [127]. The trigger of FUS cytoplasmic accumulation by XPO1 is a still controversial issue. So far, the most important link between NPC and neurodegeneration associated with FUS pathology derives from a study using transgenic flies. In this model, cytoplasmic stress granules containing FUS could be rescued by downregulating XPO1 or Nup154 (Nup155 in humans), proteins needed to export FUS from the nucleus [101, 128]. To clarify the role of NPC in FUS related-ALS and FTD, further studies aimed at exploring the NPC defects and FUS transport in brain tissues and iPSC-derived neurons are needed.

##### C9ORF72-related pathology

GGGGCC repeat expansion in the noncoding region of *C9ORF72* accounts for up to 80% of familial ALS/FTD, 20–50% of familial ALS, 5–20% of sporadic ALS, and 10–30% of FTD cases [129–131]. Of note, most patients with ALS carrying the expansions present TDP-43 pathology [95]. How C9ORF72 dysfunction leads to neurodegeneration is a matter of intensive debate. Some lines of evidence suggest a reduction in C9ORF72 transcript expression in cell lines carrying the pathological expansion, which lies in the promoter region [132]. However, *C9ORF72* knockout mice do not show neurodegeneration, raising concerns about haploinsufficiency as a primary cause of the disease [133–136]. In this view, the observation that patients who are homozygous for *C9ORF72* expansion have similar phenotypes to those who are heterozygous suggests a less important role of haploinsufficiency.

Cytoplasmic and nuclear RNA foci formed by the expanded GGGGCC repeat transcripts have been suggested to sequester RNA binding proteins and alter RNA metabolism as possible mechanisms underlying the disease [137]. The expanded hexanucleotide repeat disrupts nucleolar integrity by binding the aborted transcripts to nucleolin [138]. Of interest, nucleolar disruption also has been observed in BAC transgenic mice, *Drosophila*, human motor neurons derived from patient iPSCs, and brains [131, 139].

*C9ORF72* is also translated by an alternative mechanism to produce sense and antisense transcripts, which undergo repeat-associated non-AUG translation in all reading frames [140]. Repeat-associated non-AUG encodes five species of highly toxic DPRs, glycine-proline (GP), glycine-alanine (GA), glycine-arginine (GR), proline-alanine (PA), and proline-arginine (PR) [141, 142]. RNA foci and DPR proteins are found in the brains of *C9ORF72*-ALS/FTD patients, in human motor neurons derived from *C9ORF72*-iPSCs [143–145], and in *C9ORF72* BAC transgenic mice [139, 146, 147]. In *Drosophila*, GR and PR DPRs show toxicity, while RNA-only repeat PA or GA DPRs are not sufficient to induce degeneration [148]. In line with these findings, studies on yeast and flies confirm the toxicity of arginine-containing DPR proteins and the modifying effect of components of the NCT [149, 150]. DPRs also interact with many proteins components of the NCT [149–152]. Most proteins that interact with DPRs are low complexity domain proteins, including FUS and TDP-43 [152]. Moreover, a large-scale genetic screen approach in *Drosophila* showed an enhancement or suppression of the expansion toxicity related to several Nups, including Nup50, Nup152, TNPO1, protein shuttle mediators like Nup50, Ran, CRM1, and KPNB1, and RNA-exportins (TREX complex NXF1, Nup107, Nup160, EXOSC3, GLE1, CRM1, and others) [153]. Worth special mention are EXOSC3 and GLE1, which are strong enhancers of *C9ORF72* expansion toxicity and causative in a rare form of motor neuron disease [141].

In line with the critical role of NCT-associated proteins in C9ORF72 pathology is the finding of reduced toxicity linked to the expansion if RanGAP is overexpressed in *Drosophila* [154]. Nuclear retention of RNAs has been observed in *C9ORF72* iPSC-derived cortical neurons, indicating impairment of nuclear RNA export. Moreover, *C9ORF72* iPSC-derived neurons display a reduced nucleo–cytoplasmic ratio of Ran and RCC1, leading to defects in the NCT mechanism [150]. Recently, the mislocalization of the RNA-editing enzyme adenosine deaminase, which acts on RNA 2 (ADAR2), was found in *C9ORF72*-associated ALS/FTD, and is possibly responsible for the associated alterations in RNA processing events [155].

Taken together, these studies suggest that *C9ORF72* expansion may play distinctive roles in inducing neurodegeneration, and DPRs that lead to NCT defects is a primary pathogenic mechanism.

##### SOD1-related pathology

*SOD1* mutations are present in approximately 15% of familial and 1–3% of sporadic ALS cases [156]. Although *SOD1* was the first gene causatively linked to ALS, no widely accepted mechanisms have emerged to explain the toxicity of the mutant protein [35]. Several hypotheses have been proposed, from the impaired superoxide dismutase activity to misfolded protein aggregation and microglia activation [35]. The loss of the antioxidant function of Cu,Zn-superoxide dismutase leads to RNA oxidation and selective motor neuron degeneration [156, 157].

Some recent evidence suggests an impact of *SOD1* mutations on NCT. Mice expressing mutant *SOD1* show misregulation of different NPC components and import factors [158]. Specifically, a transgenic G93A mouse displays reduced immunoreactivity of importin-α and importin-β in the nucleus, with an increase in the cytoplasm [158]. In the same mouse model, the nuclear membrane shows irregular Nup62 staining, and Lewy body-like hyaline cytoplasmic inclusions are found in spinal motor neurons [158, 159]. Finally, irregular NM morphology was found in spinal motor neurons of ALS patients with SOD1 mutations. Taking as examples the murine models, studies aimed at investigating alterations of several Nups in cortical and spinal motor neurons of patients with *SOD1* mutations will be useful to understand whether a more complex defect of NPCs and NCT underlines the observed nuclear membrane alterations [159].

##### GLE1-related pathology

GLE1 was first identified as causative in a severe autosomal recessive lethal congenital contracture syndrome 1 and lethal arthrogryposis with anterior horn cell disease [160, 161]. Later, nonsense and missense mutations in this gene were found in ALS [162].

GLE1 is a conserved multifunctional protein that regulates gene expression, and its role has been studied in several processes, including nuclear mRNA export, translation initiation, and termination [163]. GLE1 interacts with the nucleoporin Nup155 [161], and impairment in GLE1 thus is expected to lead to NCT deficiencies. GLE1 mutations found in ALS are linked to mRNA degradation and reduced interaction with NupL2 [162]. Moreover, GLE1 is a strong enhancer of *C9ORF72* expansion–related toxicity in a *Drosophila* model [141].

#### Huntington’s disease

A consistent proportion of NCT impairment in neurodegenerative diseases is represented by the polyglutamine expansion that interferes with the normal structure and function of the NPC [164]. In HD, the CAG repeat expansion in the Huntingtin (*HTT*) gene is responsible for most of the deleterious effects that lead to death of striatum medium spiny neurons [165, 166]. Among these mechanisms, damage of polyglutamine on the neurons can be mediated by interference with NCT components, or a direct impairment of NPCs, or indirect interaction with specific Nups. An example of the former is the interaction between the N-terminal of HTT and XPO1, shown in HEK cells, which promotes shuttling between the cytoplasm and nucleus [167]. These fragments also interact with Nup TPR to export HTT from the nucleus, a mechanism that is impaired in the presence of pathological polyglutamine expansion [38]. Studies about HTT-NCT performed on iPSC-derived neurons would help to better comprehend these mechanisms and the selectivity of the disease to specific neuronal populations.

The finding of GLE1 among proteins sequestered within polyglutamine inclusions in HD suggests an indirect mechanism of HTT interference with NPCs and further establishes a link between HTT expansion and NPC defects [168]. Moreover, oxidative stress has long been held to be key to disease progression in HD [169]. Several other recent lines of evidence suggest a role for HTT and its interactions with NCP in neurodegeneration [37]. In mouse models of HD, for example, several proteins important for NCT, including RanGAP1, Nup62, and Nup88, form intranuclear inclusions that co-localize with HTT aggregates in striatal and cortical neurons [37].

A *Drosophila* model highlights the importance of antisense repeat–associated non-ATG (RAN) proteins in nuclear import [170]. These proteins accumulate in several brain regions with neuronal loss and microglia, including the striatum and white matter [170]. Of note, the pathological phenotype in *Drosophila* is enhanced with the expression of a dominant-negative form of Ran and rescued by the overexpression of Ran and RanGAP1, which seem to be neuroprotective in HD [37]. In HD patients’ iPSC-derived neurons, the MAP2 nucleo–cytoplasm ratio is significantly increased, indicating that NPCs may be damaged and dysfunctional [37]. Additional results along these lines come from neuropathological analyses of brains from patients with HD. Staining for RanGAP1 and Nup62 showed profound mislocalization in striatal neurons, consistent with the observation of selective striatal pathology in infantile bilateral striatal necrosis caused by Nup62 mutations [37].

#### Other neurodegenerative disorders with expansion repeats

Spinocerebellar ataxias include several autosomal dominant forms and one recessive form, which are caused by the expansion of CAG or CGG repeats. In spinocerebellar ataxia (SCA) 1–2–3-6-7-17 and Friedreich’s ataxia, the CAG expansions encode for polyglutamine [171]. Other forms (SCA 8–10–31-36) are not polyglutamine repeat diseases, however, and the expansions may follow an aberrant pathogenic mechanism similar to that of *C9ORF72*. In particular, various combinations of the expansions (GGGGCC-sense and GGCCCC-antisense) are improperly translated into RAN proteins, which are known to be toxic for the cell [164].

For example, Ataxin-3 enters into the nucleus through NPCs, imported by importin α/β. XPO1 and Kap α3 modulate trafficking in HEK cells, *Drosophila*, and mice. Increased cytoplasmic localization of the expanded Ataxin-3 and reduction of protein aggregates have been observed by Kap α3 knockdown and XPO1 overexpression [172, 173]. The altered interaction between Kap α3 and pathologic expanded Ataxin-3 may suggest a link between the pathogenic mechanism and the NCT, but further studies are needed.

Myotonic dystrophy type 1 (DM1) is caused by CGG repeat expansion in the 3′ untranslated region, similar to the site of expansion in SCA8. Reduced levels of Ref1, the *Drosophila* orthologue of AlyRef in mammals, exacerbates neurodegeneration in a DM1 model of *Drosophila*, suggesting a possible underlying role of an impaired RNA transport mechanism, which deserves more studies [164].

RAN proteins translation also appears to have a prominent role in the nuclear retention of transcripts in Fragile-X tremor ataxia syndrome [174]. Fragile-X tremor ataxia is caused by the premutation of *FMR1* CGG promoter expansion [175, 176], possibly indicating a pathogenic mechanism similar to that observed in HD, which is an interesting avenue of study to pursue. The protein encoded seems to be important during neural differentiation to read N6-methyladenosine modification of mRNA and promote nuclear export of methylated mRNAs [177].

Recently, impairment in the nuclear export of polyQ-expanded androgen receptor and formation of intranuclear inclusions were demonstrated in spinal and bulbar muscular atrophy [178]. In dentatorubral-pallidoluysian atrophy, which is dominantly caused by a CAG repeat in the *Atrophin1* gene, neuropathological findings show intranuclear filamentous inclusions and nuclear membrane defects in cerebellar granule cells, suggesting defects in NCT function or in NPC machinery [179, 180].

#### Recessive ataxia syndromes

*SETX* encodes the RNA/DNA helicase Senataxin. Mutations in this gene are responsible for ataxia with oculomotor apraxia (AOA) type 2 and have been linked to NCT impairment [173, 181].

In transgenic mice harboring a single *SETX* human mutation, the nuclear membrane appears damaged. The protein is less able to reach the nucleus in the presence of mutations, leading to DNA/RNA defects. In *SETX* knock-in mice, motor neurons and primary neuronal cultures show mislocalization of TDP-43 and FUS in the cytoplasm [173]. *SETX* mutations also have been reported in rare juvenile forms of ALS [182], prompting speculation about a possible link between motor neuron degeneration and TDP-43/FUS alterations.

Aprataxin, a protein involved in single-strand DNA repair breaks, is mutated in AOA type 1 [183]. Aprataxin reaches the nucleus to exert its function, binding NPCs [184]. In particular, it interacts with Kap α/β and binds the nucleoporin Aladin on the cytoplasmic side of the NPC, initial steps for import into the nucleus. This pattern suggests the importance of a correct NCT for Aprataxin to reach the nucleus and guarantees optimal DNA maintenance, which is particularly relevant for non-dividing cells.

Recently, mutations in the Nup93 were identified in an autosomal form of congenital ataxia [185]. In all of these rare forms of neurodegenerative syndromes, NPC and NCT appear to be relevant, but these preliminary observations need to be further studied.

#### Parkinson’s disease

Cytoplasmic accumulation of the protein alpha-synuclein in dopaminergic neurons of the substantia nigra is the hallmark of PD [186]. Alpha-synuclein belongs to the family of synucleins, which are proteins identified inside the nucleus, although their normal localization is in the cytoplasm. Different alpha-synuclein species bind the chromatin to induce a transcriptional modification [187]. In this role, alpha-synuclein needs phosphorylation at S129, which is specifically found in neurons of patients with an aggressive form of parkinsonism, called Lewy body dementia. Whether alpha-synuclein is transported into the nucleus is still unknown, and uncovering a possible pathological effect of alpha-synuclein aggregates on NPCs or NCT may shed light on the mechanism of PD-related neurodegeneration [187]. Oxidative damage contributes to the cascade of events leading to degeneration of highly functioning dopaminergic neurons in PD [188].

Further suggesting a possible link between altered NCT and PD, several studies have demonstrated a mislocalization of specific transcription factors in PD brains. In particular, NF-κB shows increased nuclear translocation in dopaminergic neurons of PD [189]. In addition, cytoplasmic aggregates of pCREB and the lack of the expected nuclear staining are observed in PD substantia nigra pars compacta [190]. In line with this observation, primary midbrain cultures treated with 6-hydroxydopamine show progressive accumulation of pCREB in the cytoplasm and decreased pCREB in the nucleus of dopaminergic neurons but not in nondopaminergic neurons [190].

Of note, proteins involved in recessive forms of PD have also been found to interact with Nups. Parkin, encoded by *PARK2*, is mutated in juvenile PD. It is an E3 ubiquitin ligase and specifically ubiquitinates Nup358/RanBP2, a protein fundamental to binding Ran to NPCs [191]. The role of alpha-synuclein in *PARK2*-related PD is controversial because most studies do not show alpha-synuclein accumulation in dopaminergic neurons in patients with Parkin mutations [192–194]. In addition, mice lacking one copy of Nup358/RanBP2 and treated with 1-methyl-4-phenyl-1,2,3,6-tetrahydropyridine, known to induce a PD-phenotype in animal models, have a more severe disease course and slower recovery [195, 196]. These findings suggest that NPC alterations in PD might occur regardless of alpha-synuclein accumulation. Further studies are needed to corroborate these initial observations of NCT impairment in PD, with a focus on the role of alpha-synuclein or other possible mechanisms.

#### Alzheimer’s disease

The classical pathological hallmarks of AD consist of deposition of beta-amyloid in cortical plaques, hyperphosphorylation of tau, and formation of neurofibrillary tangles, causing neuronal degeneration [197]. Initial observations suggest the presence of nuclear membrane alteration and aggregation of NPC in AD brains [198, 199]. Of interest, NTF2 accumulates in the cytoplasm of some hippocampal AD neurons regardless of the presence of tangles, and accumulation of importin-α1 has been found in CA1-hippocampal neuronal inclusions in AD brains [200]. More recently, the number of NPCs has been found to be significantly reduced in AD brains [40], and Ran-reduced expression has also been reported in postmortem tissues from AD cases [201].

The precise mechanism that leads to protein mislocalization is not clear, but one significant finding has demonstrated a link between aberrant tau accumulation and NPC impairment in AD [40]. In particular, that study provided evidence that AD-related tau disrupts nuclear pore function in AD and that Nups can trigger tau to aggregate. Specifically, one component of the NPC, Nup98, interacts with tau, facilitating its aggregation. In addition, in AD brains and tau-mutated mouse models, Nup98 is mislocalized into the cytoplasm [40]. This Nup98 mislocalization and NCT impairment can be rescued by solubilizing tau aggregates in mice. Hyperphosphorylated and mislocalized tau protein has been found also in FTD-*MAPT* neurons to lead to microtubules impairment and to damage the nuclear membrane [202, 203].

Finally, the Musashi (MSI) family of RNA-binding proteins has been recently investigated using a tau-inducible HEK model. Tau co-localizes with MSI proteins in the cytoplasm and nucleus, altering the nuclear transport of MSI and inducing structural changes to LaminB1, leading to nuclear instability [204].

#### Allgrove syndrome

A dysfunctional NCT system has been proposed to operate in triple-A syndrome, also known as Allgrove syndrome [11, 184, 205]. This rare disorder is caused by mutations in a gene that encodes the Nup Aladin. Affected fibroblasts exhibit an impaired importin-α–mediated import pathway and weak nuclear import of Aprataxin and DNA ligase I [159, 206]. As a result, cells become more susceptible to oxidative stress, accumulate damaged DNA, and undergo cell death [159]. Of interest, patients with Allgrove syndrome display a peculiar brain multisystem neurodegenerative involvement, affecting motor neurons, Purkinje cells, striatal neurons, autonomic system, peripheral nerves, and endocrine system at the same time [207]. This disease could serve as an example model to study how the same mechanism (an NPC impairment) could underlie neurodegeneration of different neuronal systems.

### Aging

In cells with a peculiar characteristic of being long-lived and non-dividing, the integrity of the genome is a fundamental issue [208]. Neurons are such cells: they do not rely on mitosis to renew their DNA and are strictly dependent for their entire lifespan on the mechanisms that guarantee the protection of DNA from external (toxins) and internal (ROS) damage. In this attempt, the machinery that provides a proper nuclear supply of DNA repair proteins (i.e., Aprataxin) and ROS scavengers must work efficiently [209]. The progressively reduced efficiency of this process and the accumulation of ROS and DNA damage lead to physiological cellular aging [210, 211].

Because the NCT is responsible for the supply role, an impaired NPC function may accelerate the alterations observed in the aging process. An example of accelerated aging from NCT deficiency is Hutchinson–Gilford progeria syndrome. This inherited laminopathy causes premature, rapid aging shortly after birth. In this syndrome, a mutant form of Lamin A leads to a dysmorphic nuclear lamina structure with the consequent alteration of the physiological RanGDP/GTP gradient, essential for a proper NCT functionality [212, 213].

Several pieces of evidence link neuronal aging with progressive NPC leaking. Aging-related progressive reduced density of NPCs has been found in the dentate gyrus of hippocampal rat neurons [214] Other studies have demonstrated altered Nups composition and turnover in aged rat neurons [215, 216]. Moreover, in iPSC-derived neurons and directly reprogrammed induced neurons from young and old humans, RanBP17 is reduced in older cells [217]. The reduced levels of this nuclear import receptor have a direct impact on the ability of aged neurons to maintain proper nuclear compartmentalization [217]. For these reasons, more elucidation of the function of Nups and their relationship with aging will significantly affect our understanding of the association of neuronal frailty with aging, opening the way to targeting the underlying molecular mechanisms.

## Conclusions

The evidence clearly indicates that NCT rely on NPCs as key factors in neuronal health and vitality. Many alterations lead to a broad spectrum of neurodegenerative patterns and to neuronal aging. The most important aspects seem to involve the maintenance of a proper RanGDP/GTP gradient and the shuttling of transcription factors and proteins involved in protein and RNA conservation. However, most of the studies investigating these mechanisms are focused on ALS and FTD models, and a deep investigation of the link of NCT, NPC and neuronal cell death is needed in other neurodegenerative diseases, especially the most common AD and PD. Moreover, to confirm the observations from animal experimental models, additional studies on human iPSC-derived neurons and neuropathological samples are needed. The reason specific neuronal subsets are variably susceptible to specific damage that leads to different phenotypes of neurodegenerative diseases remains unclear. The study of emblematic but rare diseases that display an NPC-related impairment in different neuronal types, such as Allgrove syndrome, may shed light on common mechanisms underlying these neurodegenerative processes. Moreover, targeting NCT deficiency as a common pathway of several neurodegenerative diseases and neuronal aging may represent a unique therapeutic opportunity for these incurable disorders.

## Data Availability

Not applicable.

## Abbreviations

AD: Alzheimer’s disease
ADAR2: Enzyme adenosine deaminase acting on RNA 2
ALS: Amyotrophic lateral sclerosis
AOA: Ataxia with oculomotor apraxia
CAS: Cellular apoptosis susceptibility protein
CRM1: Chromosome region maintenance 1
DM1: Myotonic dystrophy type 1
DPR: Dipeptide repeat proteins
FG: Phenylalanine-glycine
FTD: Frontotemporal dementia
GA: Glycine-alanine
GP: Glycine-proline
GR: Glycine-arginine
HD: Huntington’s disease
HTT: Huntingtin
Kaps: Karyopherins
MSI: Musashi
NCT: Nucleo–cytoplasmic transport
NES: Nuclear export signal
NLS: Nuclear localization signal
NPC: Nuclear pore complex
NTF2: Nuclear transport factor 2
NTRs: Nuclear transport receptors
Nups: Nucleoporins
NXF1: Nuclear export factor 1
PA: Proline-alanine
PD: Parkinson’s disease
PR: Proline-arginine
RAN: repeat–associated non-ATG
Ran: Ras-related nuclear protein
RCC1: Regulator of chromosome condensation 1
ROS: Radical oxygen species
SCA: Spinocerebellar ataxia
TREX: Transcription and export complex
XPO-t: Exportin-t
XPO5: Exportin-5
